# Using a constraint-based regression method for relative quantification of somatic mutations in pyrosequencing signals: a case for NRAS analysis

**DOI:** 10.1186/s13015-016-0086-4

**Published:** 2016-09-15

**Authors:** Jerome Ambroise, Jamal Badir, Louise Nienhaus, Annie Robert, Anne-France Dekairelle, Jean-Luc Gala

**Affiliations:** 1 Institut de Recherche Expérimentale et Clinique (IREC), Center for Applied Molecular Technologies (CTMA), Université catholique de Louvain, Clos chapelle-aux-champs B1.30.24, 1200 Brussels, Belgium; 2 Epidemiology and Biostatistics Department (EPID), Institut de Recherche Expérimentale et Clinique (IREC), Université catholique de Louvain, 1200 Brussels, Belgium

**Keywords:** Pyrosequencing, somatic, AdvISER-PYRO-SMQ, Sparse representation

## Abstract

**Background:**

Pyrosequencing Allele Quantification (AQ) is a cost-effective DNA sequencing method that can be used for detecting somatic mutations in formalin-fixed paraffin-embedded (FFPE) samples. The method displays a low turnaround time and a high sensitivity. Pyrosequencing suffers however from two main drawbacks including (i) low specificity and (ii) difficult signal interpretation when multiple mutations are reported in a hotspot genomic region.

**Results:**

Using a constraint-based regression method, the new AdvISER-PYRO-SMQ algorithm was developed in the current study and implemented into an R package. As a proof-of-concept, AdvISER-PYRO-SMQ was used to identify a set of 9 distinct point mutations affecting codon 61 of the *NRAS* oncogene. In parallel, a pyrosequencing assay using the Qiagen software and its AQ module was used to assess selectively the presence of a single point mutation (*NRAS*$c.182A>G$ - Q61R-1) among the set of codon 61 mutations, and to analyze related pyrosequencing signals. AdvISER-PYRO-SMQ produced a lower limit of blank (0 %) than the AQ module of Qiagen software (5.1 %) and similar limit of detection were obtained for both software (5.6 vs 4.8 %). AdvISER-PYRO-SMQ was able to screen for the presence of 9 distinct mutations with a single pyrosequencing reaction whereas the AQ module was limited to screen a single mutation per reaction.

**Conclusion:**

Using a constraint-based regression method enables to analyze pyrosequencing signal and to detect multiple mutations within a hotspot genomic region with an optimal compromise between sensitivity and specificity. The AdvISER-PYRO-SMQ R package provides a generic tool which can be applied on a wide range of somatic mutations. Its implementation in a Shiny web interactive application (available at https://ucl-irec-ctma.shinyapps.io/Pyrosequencing-NRAS-61/) enables its use in research or clinical routine applications.

**Electronic supplementary material:**

The online version of this article (doi:10.1186/s13015-016-0086-4) contains supplementary material, which is available to authorized users.

## Background

Pyrosequencing is a cost-effective DNA sequencing technique which is based on pyrophosphate release during nucleotide incorporation [1]. The four possible nucleotides are sequentially dispensed in a predetermined order. The first chemi-luminescent signal produced during nucleotide incorporation is detected by a charge-coupled device camera in the pyrosequencer and displayed in a pyrogramTM. Pyrosequencing has many applications, including short sequence analysis (SQA mode), SNP genotyping (SNP mode), quantification of CpG methylation (CpG mode), and allele quantification (AQ mode). Allele quantification is especially applied to detect and quantify somatic mutations within tumor samples. Accordingly, a dispensation order has first to be defined, using the software developed by the pyrosequencer manufacturer (Qiagen, Hilden, Germany). Usually, the selected dispensation order produces, at a specific position, a peak height which is proportional to the ratio “targeted somatic mutations/wild-type alleles” from the sample. Consequently, this specific position shows no signal (i.e. a peak height near 0) with a wild-type sample. When an unknown sample is processed, the AQ module of the Qiagen software divides the specific peak height intensity by a normalization factor which reflects the global pyro-signal intensity in order to estimate the ratio mutated/wild-type alleles within the sample.

Using the pyrosequencing for allele quantification is straightforward when a unique somatic mutation is targeted (e.g. $c.12A>C$). When a hotspot genomic region has to be analyzed (i.e. a short genomic region where multiple somatic mutations are reported as is the case with the *NRAS* oncogene where as many as nine different clinically significant point mutations are spread over codon 61), the standard AQ module cannot be used for analyzing the pyro-signal. Consequently, specific kits and plug-in software solutions were developed by the pyrosequencer manufacturer to enable the assessment of these multiple mutations through single pyrosequencing experiments. However, theses kits and plug-in software solutions are currently restricted to a limited number of well-defined genomic regions such as *KRAS*, *BRAF* and *EGFR* oncogenes. Moreover, these kits are expensive and are restricted to the pyrosequencing PyroMark Q24 instrument and can not be used with a Pyromark Q96 system.

In that context, Shen et al. developed a pyrosequencing data analysis software [2] dedicated to hotspot regions in *KRAS*, *BRAF* and *EGFR* oncogenes. Unfortunately, this software which was not distributed, was designed as a “working draft” still requiring a long and elaborated process of fine-tuning [2]. Skorokhod et al. also developed an algorithm to analyze the BRAF mutational status by constructing an elaborate decision tree based on successive ‘IF’ operators [3]. For additional hotspot genomic regions, new solutions should therefore be considered. A first would be to elaborate a home-made system requiring sophisticated manual process, but this does not prevent the risk of human errors [2]. A second solution would be to perform a pyrosequencing reaction for each somatic mutation of interest within the hotspot genomic region. However this second solution increases costs and turnaround time proportionally to the number of targeted somatic mutations. Moreover, given the limited amount of DNA that can be extracted from formalin-fixed paraffin-embedded (FFPE) samples, multiplying pyrosequencing reactions on the same sample is often technically impossible.

Despite the difficulty of interpreting pyro-signals when hotspot genomic regions are analyzed, pyrosequencing remains a useful and widely accessible analytical method presenting several advantages among which speed and cost-effectiveness. Moreover, when compared to Sanger sequencing, pyrosequencing consistently discloses a higher sensitivity enabling the detection of a lower percentage of mutated alleles in the sample. While the detection of a somatic mutation using the Sanger sequencing requires 20 % mutated tumor cells, it can be achieved by pyrosequencing with as few as 5 % mutated cells [2, 4]. In a recent study where pyrosequencing technology was compared with four other molecular methods (i.e. high resolution melting analysis, next generation sequencing, immunohistochemistry, and Sanger Sequencing) for the detection of p.V600E and non-p.V600E *BRAF* mutations, pyrosequencing showed the highest sensitivity (down to 5 % allele frequency) while showing the lowest specificity [5]. Lack of specificity observed with pyrosequencing is partially attributable to the presence of non-specific peak heights due to background noise and artifacts [2, 3].

In the present study, a constraint-based regression method was developed in order to tackle both major drawbacks of allele quantification using pyrosequencing: (i) a lack of specificity and (ii) difficult signal interpretation in case of multiple mutations in a short and well-defined genomic region (i.e. a hotspot). This constraint-based regression method was implemented in the new AdvISER-PYRO-SMQ algorithm that enables to obtain a sparse representation of the pyro-signal. Sparse representation, constraint-based, and penalized regression methods have received a lot of attention in recent years [6]. These methods were applied, inter alia, on gene expression data to classify tumors [7], on miRNA and mRNA expression data for glioblastoma subtyping [8], and on single nucleotide polymorphisms (SNP) and functional magnetic resonance imaging (fMRI) voxels to discriminate between schizophrenia cases and controls [6]. Regarding pyrosequencing analysis, sparse representation via constraint-based regression method was recently used to develop three complementary software solutions: (i) the AdvISER-PYRO software for analyzing low and complex signals resulting from samples including several mycobacteria [9], (ii) the AdvISER-M-PYRO software for analyzing overlapping pyro-signals generated from multiplex reactions conducted on mono-allelic genes in bacteria [10], and (iii) the AdvISER-MH-PYRO software for analyzing overlapping pyro-signals generated from multiplex reactions to genotype bi-allelic human SNP [11].

As a proof-of-concept, the new AdvISER-PYRO-SMQ software was applied in the present study to detect multiple mutations (N = 9) in codon 61 of the *NRAS* oncogene. *NRAS* mutation status is known to impact survival time of patients with melanoma [12] and it is used as a prognostic and predictive marker in metastatic colorectal cancer [13]. The specific somatic mutation *NRAS*$c.182A>G$ (Q61R-1 variant) was analyzed in order to compare Limit of Blank (LoB) and Limit of Detection (LoD) obtained with the new software versus a pyrosequencing assay developed with the AQ module of PyroMark Q96 2.5.8 software.

## Methods

### Dilution series

In order to compare the LoB and LoD obtained both with AQ module of PyroMark Q96 2.5.8 software and the new AdvISER-PYRO-SMQ software, dilution series (N = 3) were carried out and calibration curves were computed from data recorded with each dilution series and with both software solutions.

In a first step, two 131-bp nucleotide sequences [$gBlock^{TM}1$ and $gBlock^{TM}2$ Gene Fragments (Integrated DNA Technologies, Leuven, Belgium)] were synthesized. Both gBlocks included the codon 61 of the *NRAS* gene with the first gBlock (gBlock1) matching the wild-type sequence while the second gBlock (gBlock2) matching a selected *NRAS* mutant variant (*NRAS*$c.182A>G$- Q61R-1). Both synthetic olignonucleotide sequences included the pyrosequencing primer (TCATGGCACTGTACTCTT), the forward PCR primer (TGAAACCTGTTTGTTGGACATACT), and the reverse PCR primer (CCGCAAATGACTTGCTATTATTG). Samples with gBlock2 were serially diluted with gBlock1 to reach the following proportions of gBlock2: 50, 10, 5, 2.5, 1.25 and 0 %. Three dilution series and six replicate samples per concentration were prepared. Three of the 6 replicate samples were pyrosequenced with the dispensation order defined by PyroMark Q96 2.5.8 software and analyzed with the AQ module of the same software whereas the three remaining samples were pyrosequenced with a dispensation order defined by SENATOR [10] and analyzed with the new AdvISER-PYRO-SMQ algorithm.

### Pyrosequencing

Except for the dispensation order which was modified for half of the samples, pyrosequencing was carried out according to manufacturer’s protocol. Briefly, PCR was carried out in a 50 μL reaction mixture containing 5 μL of the extracted DNA (0.06 ng/μL), 5 μL of a PCR buffer (100 mM Tris-hydrochloride, and 500 mM potassium chloride, pH 8.3), 4.5 μL of MgCl_2_ 25 mM, 0.2 μL of AmpliTaq Gold®DNA Polymerase 5U/μL (AmpliTaq Gold DNA Polymerase kit from Applied Biosystems, Austin, USA), 4 μL of dNTPs 2.5 mM (dNTPs: dATP, dCTP, dGTP, dTTP Li-salts from Roche Diagnostics GmbH, Mannheim, Germany) and 2 μL of forward and reverse PCR primers 10pm/μL (Eurogentec, Liege, Belgium).

Amplification was performed in a 2720 Thermal Cycler (Applied Biosystems) using the following conditions: 95 °C for 5 min, followed by 40 cycles with denaturation at 95 °C for 40 s, annealing at 59 °C for 40 s, and extension at 72 °C for 80 s, with a final elongation step at 72 °C for 7 min. Pyrosequencing was then carried out with a PyroMark Q 96 ID Sequencer from Qiagen (Hilden, Germany) on PCR products, using the pyrosequencing primer, enzymes and substrate (PyroMark Gold®Q96 Reagents kit, Qiagen) according to the manufacturer’s protocol. Each PCR and pyrosequencing reaction included negative and positive controls.

### Pyro-signal analysis using Allele Quantification module of Qiagen

For each dilution series (N = 3), and each proportion (N = 6), three replicates were pyrosequenced with the dispensation order generated with the PyroMark Q96 2.5.8 software. This dispensation order was designed in order to target the *NRAS*$c.182A>G$ (Q61R-1) mutated variant. Pyro-signals were all analyzed using the Allele quantification (AQ) module of the same software. Percentages of *NRAS*$c.182A>G$ (Q61R-1) mutated alleles were recorded and used to compute a single calibration curve for each dilution series. The LoB and LoD were then deduced from each calibration curve. The LoB was computed as the highest percentage of somatic mutation expected to be computed by the software when replicates of blank samples (i.e. 100 % WT-0 % Q61R-1) are tested [14]. Conversely, the LoD was computed as the lowest percentage of somatic mutation likely to be reliably distinguished from the LoB and at which detection was feasible [14]. The LoD was therefore set at the intersection between the LoB and the prediction interval of the calibration curve.

### Pyro-signal analysis using AdvISER-PYRO-SMQ

For each dilution series (N = 3), and each concentration (N = 6), three replicates were pyrosequenced with a dispensation order generated by SENATOR, as previously recommended [10]. The analysis of all pyro-signals was then carried out with AdvISER-PYRO-SMQ. It is worth noting that in this paper, a pyro-signal is defined as the global pattern integrating all successive peak heights and corresponds therefore to a vector whose length n equals the number of dispensed nucleotides (n = 12 in the current application, see "Results'' section). The development of this algorithm included the three following steps.

Firstly, a standardized learning dictionary was created including a uniplex theoretical pyro-signal for each of the 10 possible Unique Nucleotide Sequence (UNS) of the current application [WT, *NRAS*$c.181C>G$ (Q61E), *NRAS*$c.181C>A$ (Q61K), *NRAS*$c.182A>T$ (Q61L-1), *NRAS*$c.182A>C$ (Q61P), *NRAS*$c.182A>G$ (Q61R-1), *NRAS*$c.182_183AA>TG$ (Q61L-2), *NRAS*$c.182_183AA>GG$ (Q61R-2), *NRAS*$c.183A>C$ (Q61H-1), *NRAS*$c.183A>T$ (Q61H-2)]. Aside of the 10 theoretical pyro-signals, 6 experimental signals of the WT variants were generated by pyrosequencing gBlock1. These experimental signals were standardized by dividing all peak heights by the corresponding unitary peak height, as previously recommended [9], and compiled with theoretical pyro-signals within the dictionary. The dictionary consisted therefore in a matrix with 16 columns (i.e. 1 column for each pyro-signal) and 12 rows (i.e. 1 row for each dispensed nucleotide).

In a second step, each pyro-signal (vector y) of length n (n = 12) was analyzed with AdvISER-PYRO-SMQ software. With this software, the pyro-signal y is modelled as a sparse linear combination of the p (p = 16) pyro-signals of length n (n = 12) from the dictionary using a constraint-based regression method. The least absolute shrinkage and selection operator (lasso) method [15] uses a L1-norm constraint on the coefficient vector $\beta$ and the issue is therefore to find a vector $\beta$ of length p (p = 16) minimizing the following function:

1 $$\begin{aligned} \sum ^{n}_{i=1}\left(y_{i}-\sum ^{p}_{j=1}\beta _{j}x_{ij}\right)^{2} \end{aligned}$$

with the following constraint on the sum of the absolute value of each element within the $\beta$ coefficient vector (i.e. a L1-norm constraint on $\beta$) :

2 $$\begin{aligned} \sum ^{p}_{j=1} \left| \beta _{j} \right| \le s \end{aligned}$$

Solving this constraint-based minimization problem is equivalent to minimizing the following penalized regression equation.

3 $$\begin{aligned} \sum ^{n}_{i=1}\left(y_{i}-\sum ^{p}_{j=1}\beta _{j}x_{ij}\right)^{2} + \sum ^{p}_{j=1} \lambda \left| \beta _{j}\right| \end{aligned}$$

where $y_{i}$ is the ith element of the y pyro-signal, $x_{ij}$ is ith element of the jth pyro-signal from the dictionary, $\left| \beta _{j}\right|$ is the absolute value of the jth coefficient from the $\beta$ coefficient vector, and $\lambda$ is a shrinkage parameter. For every $\lambda$ value in equation 3, there is a bound parameter s in equation 2 yielding the same solution. Selecting $\lambda = 0$, or equivalently a sufficiently large value of s, yields to the standard least square solution. Increasing the value of $\lambda$, or equivalently a decreasing the value of s, increases the sparsity of the solution [16].

While a unique $\lambda$ parameter was applied for all signals from the dictionary in previous applications of AdvISER-PYRO [9], AdvISER-M-PYRO [10], and AdvISER-MH-PYRO [11], low shrinkage parameters (i.e $\lambda = 0$) and higher shrinkage parameters (i.e. $\lambda = 50$) were applied on pyro-signals corresponding to the WT sequence and to the other variants, respectively, in the current AdvISER-PYRO-SMQ application. It is worth noting that the shrinkage value which is selected for somatic mutation can be tuned to improve specificity (with higher values i.e. $\lambda = 100$) or sensitivity (with lower values i.e. $\lambda = 5$). Because the signal contribution of each UNS should have a positive value, an additional constraint was implemented through the ‘positive’ parameter of the penalized function the corresponding R package [17]. In this package, the elements of the $\beta$ coefficient vector are estimated through an algorithm based on a combination of gradient ascent optimization with the Newton–Raphson algorithm [18]. After model estimation, the sum of regression coefficients corresponding to each UNS was computed and recorded as the UNS contribution to the signal.

While not implemented with the previous AdvISER-PYRO and AdvISER-M-PYRO versions [9, 10], the third step of the new AdvISER-PYRO-SMQ algorithm involved to select the most likely somatic mutation and to quantify the percentage of mutated allele. Accordingly, the selection of the two main contributing UNS (i.e. WT and one selected mutation) was carried out by iteratively removing from the dictionary the signals associated with the lowest UNS contribution. The quantification of the selected somatic mutation was computed as the relative contribution of the selected somatic to the global signal. The relative quantification was computed for each sample and was recorded in order to compute one calibration curve for each dilution series. The LoB and LoD were then deduced from each calibration curve.

When a pyro-signal is analyzed by the software, a correlation coefficient (r) is computed between predicted values of the penalized regression model and peak heights of the observed pyro-signal (i.e. the elements of the y vector). Considering that a low correlation coefficient is indicative of a discrepancy between the observed pyro-signal y and the selected combination of pyro-signals from the dictionary, this coefficient was used to assess the global confidence of the predicted UNS combination.

## Results

### Selection of the nucleotide dispensation order

As this pyrosequencing experiment was carried out using reverse primers, the reverse complementary sequence was computed for each UNS (Table 1). SENATOR was then used to select a dispensation that enables to differentiate all UNSs of interest for the current application (Table 1).

**Table 1:** List of all unique nucleotide sequence (UNS) of interest in the current application

Variant name	Unique nucleotide sequence	Reverse complementary
WT	AGCTGGA**CAA**G	C**TTG**TCCAGCT
*NRAS* $c.181C>G$ (Q61E)	AGCTGGA**GAA**G	C**TTC**TCCAGCT
*NRAS* $c.181C>A$ (Q61K)	AGCTGGA**AAA**G	C**TTT**TCCAGCT
*NRAS* $c.182A>T$ (Q61L-1)	AGCTGGA**CTA**G	C**TAG**TCCAGCT
*NRAS* $c.182A>C$ (Q61P)	AGCTGGA**CCA**G	C**TGG**TCCAGCT
*NRAS* $c.182A>G$ (Q61R-1)	AGCTGGA**CGA**G	C**TCG**TCCAGCT
*NRAS* $c.182\_183AA>TG$ (Q61L-2)	AGCTGGA**CTG**G	C**CAG**TCCAGCT
*NRAS* $c.182\_183AA>GG$ (Q61R-2)	AGCTGGA**CGG**G	C**CCG**TCCAGCT
*NRAS* $c.183A>C$ (Q61H-1)	AGCTGGA**CAT**G	C**ATG**TCCAGCT
*NRAS* $c.183A>T$ (Q61H-2)	AGCTGGA**CAC**G	C**GTG**TCCAGCT

Nucleotides of codon 61 are in bold characters

**Fig. 1:**
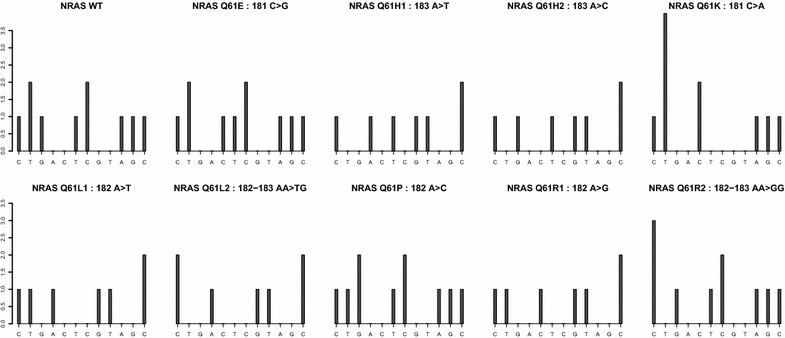
Pyro-signals corresponding to each unique nucleotide sequence (UNS) of interest in the current application and according to the selected dispensation order as defined by the SENATOR algorithm

A nucleotide dispensation order with 12 nucleotides (CTGACTCGTAGC) was selected. This dispensation order generated theoretical uniplex pyro-signals with low pairwise correlation coefficients (Fig. 1), avoiding collinearity between signals which are contained in the dictionary. These pyrosignals were used as predictors in the penalized regression models within the AdvISER-PYRO-SMQ algorithm. It is worth noting that the selected dispensation order covers three *NRAS* codons (59, 60 and 61).

### Pyro-signal processing using AQ module of PyroMark Q96 2.5.8 software

**Fig. 2:**
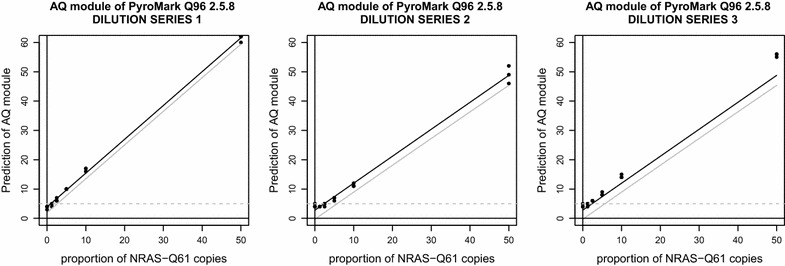
Calibration curves generated from the predictions of the AQ module of the PyroMark Q96 2.5.8 software

Pyro-signals from each dilution series (N = 3) were analyzed with AQ module of the PyroMark Q96 2.5.8 software and the resulting quantifications were used to compute one calibration curve for each dilution series (Fig. 2). LoB and LoD corresponding to the each dilution series are given in Table 2. Blank samples (i.e. 100 % WT-0 % Q61R-1) produced non-specific peaks which led to false-positive detection of Q61R1 allele ranging from 3 to 5 %. When a standard decision threshold of 5 % was considered [2, 5], 22 % (2/9) of these blank samples still produced false positive results. Samples with a predicted proportion of about 8 % were reliably distinguished from the LoB, corresponding to a Q61R1 allele proportion of about 5 % (i.e. $LoD \approx 5\,\%$).

**Table 2:** Limit of blank and limit of detection obtained from the three calibration curves produced with the AQ module of the PyroMark Q96

	Dilution series 1 (%)	Dilution series 2 (%)	Dilution series 3 (%)	Average (%)
LoB	4.6	5.3	5.3	5.1
LoD	2.7	5.8	5.8	4.8

### Pyro-signal processing using Adviser-PYRO-SMQ

**Fig. 3:**
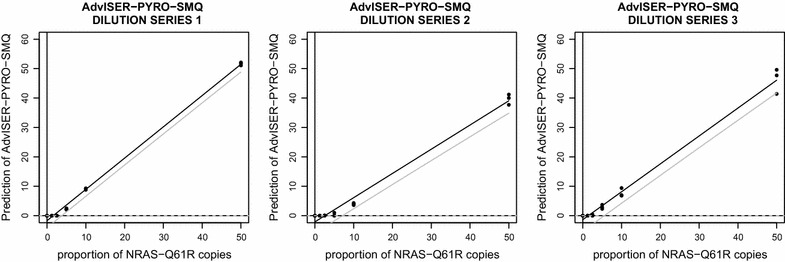
Calibration curves generated from the predictions of the AdvISER-PYRO-MSQ software

Calibration curves obtained from the interpretation of AdvISER-PYRO-SMQ on pyro-signals from each dilution series are displayed in Fig. 3.

**Table 3:** Limit of blank and limit of detection obtained from the three calibration curves produced with the new AdvISER-PYRO-MSQ software

	Dilution series 1 (%)	Dilution series 2 (%)	Dilution series 3 (%)	Average (%)
LoB	0.0	0.0	0.0	0.0
LoD	3.9	7.2	5.6	5.6

The LoB and LoD corresponding to the three calibration curves are given in Table 3. For all pure WT samples, a predicted proportion of Q61R1 allele of 0 % was systematically obtained, resulting into a LoB of 0 %. Irrespectively of the dilution series, the LoD obtained with Adviser-Pyro-SMQ was similar to the results obtained with PyroMark Q96 2.5.8 software.

### Impact of shrinkage parameter on specificity and sensitivity

Results presented in the previous section were obtained with a low shrinkage parameter ($\lambda = 0$) which was applied on WT pyro-signals from the dictionary and with a single shrinkage parameter (i.e. $\lambda =50$) which was applied to all type of mutations. As explained before, the latter shrinkage parameter can be modified for each element of the standardized learning dictionary. As demonstrated in this section, this specific feature of the new Adviser-Pyro-SMQ algorithm (compared to previous AdvISER-PYRO, AdvISER-M-PYRO, and AdvISER-MH-PYRO applications) is highly relevant for somatic mutation quantification because it impacts the trade-off between sensitivity and specificity.

**Fig. 4:**
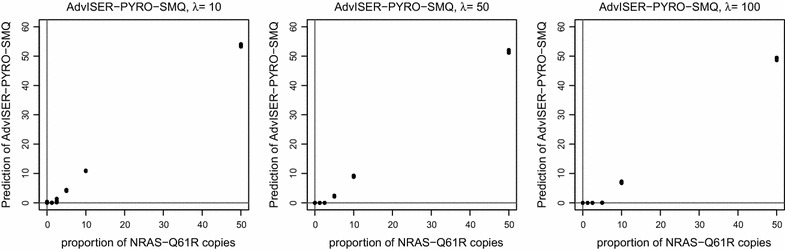
Calibration curves obtained with the dilution series n°1 and with three distinct shrinkage lambda parameters (3, 30, 100) applied on pyro-signals corresponding to the *NRAS*-61 mutations

In this context, all pyro-signals from the first dilution series were analyzed with three different shrinkage parameters ($\lambda$ = 5, 50 and 100) applied on the Q61R-1 mutation. Shrinkage parameters applied on the WT ($\lambda = 0$) and other mutations ($\lambda = 50$) were kept constant. As shown in Fig. 4, a lower shrinkage parameter ($\lambda = 5$) decreased the risk of false-negative result (i.e. improved sensitivity) for samples with a small proportion of Q61R-1 alleles. But consequently, this low shrinkage parameter increased the risk of false-positive result (i.e. decreased specificity). Conversely, a higher shrinkage parameter ($\lambda = 100$) improved the specificity while affecting the sensitivity. Indeed, all samples with a low number of mutated cells (Q61R < 10 %) were identified as containing only wild-type alleles when a higher shrinkage parameter ($\lambda = 100$) was used.

### Use and illustration of AdvISER-PYRO- SMQ

**Fig. 5:**
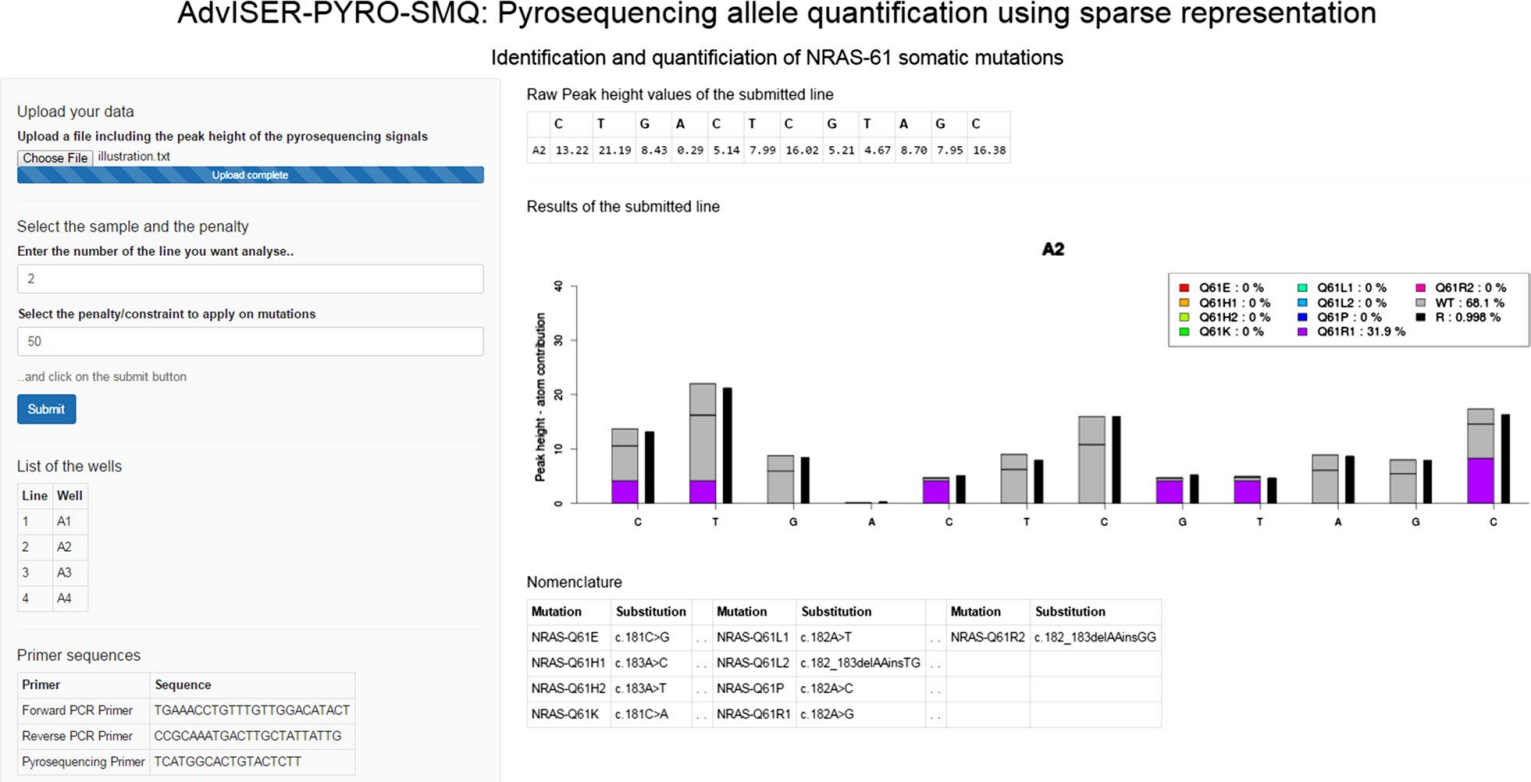
The AdvISER-PYRO-SMQ software is implemented in a Shiny application available at https://ucl-irec-ctma.shinyapps.io/Pyrosequencing-NRAS-61/

AdvISER-PYRO-SMQ was implemented in an R package (Additional file 1) which can be applied to analyze pyro-signals generated for the detection and quantification of a broad range of somatic mutations. As it is not always feasible for all laboratories to use R commands in order to analyze pyro-signals from clinical routine applications, we also developed a Shiny application (shown in Fig. 5 and available at https://ucl-irec-ctma.shinyapps.io/Pyrosequencing-NRAS-61/) to demonstrate that the available R package can be converted into a web interactive application, facilitating its use in research or clinical routine applications.

**Fig. 6:**
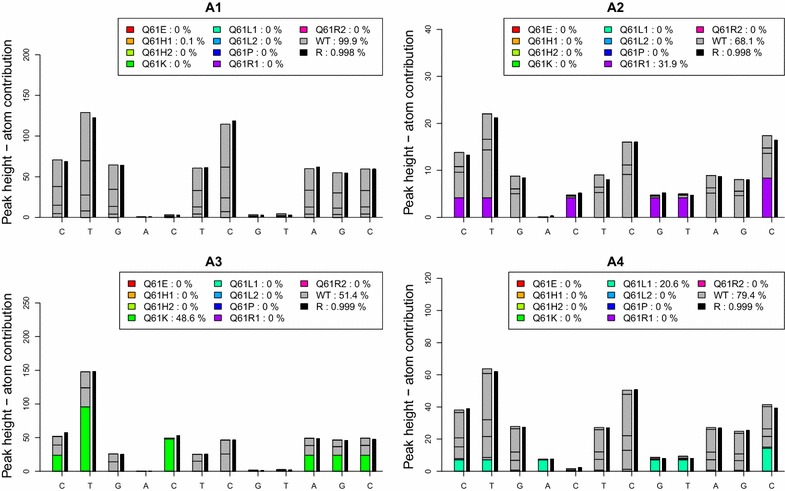
Example of four pyro-signal identifications using AdvISER-PYRO-SMQ. The pyro-signals generated by the pyrosequencer are represented by *vertical black lines*. The contribution of each WT pyro-signal within the dictionary is represented by a *dark gray box* while the contribution of each somatic mutation signal within the dictionary is represented by a specific color (e.g. *light green* for Q61K somatic mutation in well A3)

In this Shiny application, the user must upload the raw peak dataset extracted from the Pyrosequencing machine. A raw peak dataset including 4 different pyro-signals obtained from pyrosequencing analysis of FFPE samples is available (Additional file 2). In this dataset, each line corresponds to one sample and each column corresponds to a nucleotide dispensation. The user must then select the line to be analyzed and the penalty/shrinkage paramater before pushing on the submit button to obtain the result. Figure 6 illustrates the results obtained with AdvISER-PYRO-SMQ when applied on the 4 pyro-signals from the available dataset. While no somatic mutation was detected in sample A1, 31.9 % of Q61R1, 48.6 % of Q61K, and 20.6 % of Q61L1 somatic mutations were detected in A2, A3, and A4 samples, respectively. For each pyro-signal, peak heights of >20 relative fluorescence units (RLU) were observed and the correlation coefficient (r) between predicted values of the penalized regression model and the 12 values of the pyrosequencing signal was >0.995. Both factors have to be considered to assess the validity of signal interpretation. It is of note that a low correlation coefficient would be obtained with a sample presenting a new mutation not yet included within the dictionary. A tumor sample with a new mutation of exon 59 of *NRAS* oncogene (as present in exon 59 of *KRAS* oncogene) would therefore produce a low correlation coefficient, allowing the operator to detect this unusual sample.

## Discussion

Pyrosequencing Allele Quantification is a cost-effective DNA sequencing method that can be used for detecting somatic mutations in FFPE samples. This method displays a low turnaround time and a high sensitivity. Pyrosequencing suffers from drawbacks related to the analysis and interpretation of the pyro-signals.

The first disadvantage is the low specificity characterizing the Allele Quantification (AQ) module of the Qiagen software. Indeed, when the usual decision threshold of ≥5 mutated alleles for a “mutation-positive” sample is chosen [2, 5], a low specificity affects the current NRAS application. In that respect, analyzing blank samples with the AQ module produced false-positive (2/9, 22 %) predictions, resulting into a lack of specificity. While Gblock samples produced pyro-signals with high signal-to-noise ratio in the current study, pyro-signals generated from FFPE clinical samples could produce noisy pyro-signals which would further alter the specificity of the analysis. While the specificity of the AQ module can theoretically be improved by increasing the decision threshold (i.e. from 5 to >5), this would imply to compute a specific threshold for each type of somatic mutation by computing the corresponding calibration curve. Determining the mutational status would therefore require comparing each predicted percentage to a specific threshold.

In the current study, a constraint-based regression method was used to quantify somatic mutation from pyro-signals. This method was implemented in the new AdvISER-PYRO-SMQ algorithm which predicted the absence of mutated alleles in all blank samples. Even with a low decision threshold (e.g. 1 %), analyzing pyro-signals with AdvISER-PYRO-SMQ produced highly specific result. Moreover, shrinkage parameters can be adjusted in this new algorithm, a useful feature enabling the users to improve either the specificity or the sensitivity.

A second disadvantage of pyrosequencing is related to the interpretation of the pyro-signal when several different mutations can affect the same short genomic region (i.e. a hotspot). Analyzing such hotspot regions requires either to multiply the number of pyrosequencing reactions to analyze with the standard AQ module of Qiagen, or to develop home-made system requiring sophisticated manual process which do not prevent the occurence of human errors.

In the current study, it was demonstrated how AdvISER-PYRO-SMQ can target multiple somatic mutations in the codon 61 of NRAS. The pyro-signals were automatically interpreted by the software which produces a simple output that can directly be transmitted to the physician in charge of the patient.

## Conclusion

AdvISER-PYRO-SMQ is a generic software which allows the detection of a wide range of somatic mutations including standard point mutations but also multiple mutations within a single genomic region. As demonstrated here, this new algorithm can also be implemented in an interactive web application, facilitating its use in research or clinical routine applications.

## Additional files

10.1186/s13015-016-0086-4 Source code of AdvISER-PYRO-SMQ. The source code for the new R package which implements the AdvISER-PYRO-SMQ algorithm.
10.1186/s13015-016-0086-4 Dataset NRAS-61.txt. A dataset to illustrate the use of the Shiny web interactive application. The dataset includes the pyro-signals (i.e.the raw peak heights) corresponding to four samples (A1, A2, A3, and A4).

## Abbreviations

AQ: Allele Quantification
FFPE: formalin-fixed paraffin-embedded
LoB: Limit of Blank
LoD: Limit of Detection
UNS: Unique nucleotide sequence
WT: wild type
